# Whole-body MRI in pediatric patients with cancer

**DOI:** 10.1186/s40644-017-0107-7

**Published:** 2017-02-10

**Authors:** Marcos Duarte Guimarães, Julia Noschang, Sara Reis Teixeira, Marcel Koenigkam Santos, Henrique Manoel Lederman, Vivian Tostes, Vikas Kundra, Alex Dias Oliveira, Bruno Hochhegger, Edson Marchiori

**Affiliations:** 1 Department of Imaging, AC Camargo Cancer Center, Rua Prof. Antônio Prudente, 211, Liberdade, São Paulo/SP, 01509-010 Brazil; 2 Universidade Federal do Vale do São Francisco (UNIVASF), Av. José de Sá Maniçoba, Petrolina, PE 56304-917 Brazil; 3 Department of Imaging, AC Camargo Cancer Center, Rua Prof. Antônio Prudente, 211, Liberdade, Sao Paulo/SP 01509-010 Brazil; 4 Division of Radiology, Department of Internal Medicine, Ribeirao Preto Medical School, University of Sao Paulo, Av. Bandeirantes, 3900, Ribeirao Preto/ SP, 14049-090 Brazil; 5 Universidade Federal de São Paulo, Departamento de Diagnóstico Por Imagem, Disciplina de Diagnóstico por Imagem em Pediatria, Rua Napoleão de Barros, 800, Vila Clementino, Sao Paulo/SP 04024002 Brazil; 6 Universidade Federal de São Paulo, Centro de Diagnóstico por Imagem do Instituto de Oncologia Pediátrica e Médica Radiologista do Centro de Diagnóstico por Imagem do Instituto de Oncologia Pediátrica, Rua Napoleão de Barros, 800, Vila Clementino, Sao Paulo/SP 04024002 Brazil; 7 Department of Diagnostic Radiology, The University of Texas MD Anderson Cancer Center, 1515 Holcombe Blvd, Houston, TX 77030 USA; 8 Department of Radiology, Universidade Federal de Ciências da Saúde de Porto Alegre, Rua Professor Anes Dias, 285, Centro Histórico, Porto Alegre/RS 90020-090 Brazil; 9 Department of Radiology, Universidade Federal do Rio de Janeiro, Rua Thomaz Cameron, 438, Valparaíso, Petrópolis/RJ 25685-129 Brazil

**Keywords:** Neoplasm, Pediatrics, Magnetic resonance imaging, Whole body MRI, Whole body imaging

## Abstract

Cancer is the leading cause of natural death in the pediatric populations of developed countries, yet cure rates are greater than 70% when a cancer is diagnosed in its early stages. Recent advances in magnetic resonance imaging methods have markedly improved diagnostic and therapeutic approaches, while avoiding the risks of ionizing radiation that are associated with most conventional radiological methods, such as computed tomography and positron emission tomography/computed tomography. The advent of whole-body magnetic resonance imaging in association with the development of metabolic- and function-based techniques has led to the use of whole-body magnetic resonance imaging for the screening, diagnosis, staging, response assessment, and post-therapeutic follow-up of children with solid sporadic tumours or those with related genetic syndromes. Here, the advantages, techniques, indications, and limitations of whole-body magnetic resonance imaging in the management of pediatric oncology patients are presented.

## Background

Cancer is currently the leading cause of natural death in the pediatric populations of developed countries [1]. However, the cure rates for cancers are greater than 70% in some cases when a cancer is diagnosed in its early stages. To increase the cure rates for cancer patients, diagnostic and therapeutic advances are needed. To select the most appropriate treatment for a child with cancer, the type, location, and staging of the tumour should be completely assessed [2–4]. Ideally, imaging protocols should be rapid, provide high quality images, have a low radiation, and provide clinically significant information [5–7]. In addition, every effort should be made to avoid redundant examinations that do not provide additional information relevant to therapeutic decision making [8]. There are many imaging modalities that are currently used to characterise the extent of local and distant disease. For example, ultrasonography, computed tomography (CT), magnetic resonance imaging (MRI), metaiodobenzylguanidine (MIBG) scans, and bone scintigraphy (BS) are most frequently performed [8, 9]. However, modalities that deposit radiation, such as BS and CT, should be used with caution in pediatric patients due to the risk of complications, including the risk of developing secondary malignancies.

Positron emission tomography/computed tomography (PET/CT) has recently been used in children with cancer because it provides whole-body (WB) coverage and information regarding the metabolic stage of tumours. Regarding the latter, an intravenous injection of the radiopharmaceutical, ^18^F-fluorodeoxyglucose (^18^F-FDG), allows regions of abnormal glucose metabolism to be detected with hybrid systems such as PET/CT and correlated with possible morphological changes on anatomic images [10, 11]. PET/CT also plays an important role in tumour staging, in assessing response to treatment, and can potentially predict treatment success in certain oncology settings [12]. Operational limitations of PET/CT include its restricted availability to specialised centers in many countries; the short half life of ^18^F which requires prompt delivery and use [13]; and the radiation burden to a patient.

In contrast, MRI can provide exquisite anatomic detail and functional information without radiation to patients. In addition, technological advances, particularly in the development of fast imaging sequences, allow MRI to provide WB coverage in a reasonable time frame [14, 15]. Thus, with the fusion of morphological sequences and functional techniques, such as diffusion-weighted imaging (DWI) and WB morphological/functional mapping, relevant information regarding disease activity can be obtained [16, 17]. The aim of this review is to discuss the advantages, techniques, indications, and limitations of WB MRI in evaluations of pediatric patients with cancer.

## Main Text

### Advantages of WB MRI

WB MRI provides a single examination of the entire body without the use of ionizing radiation. In addition to the excellent contrast and spatial resolution of WB MRI, functional information is obtained which improves the capacity of this method to differentiate normal tissues from pathological tissues. MRI equipment is often available at both large and small centers, thereby facilitating the implementation of more advanced studies, such as MRI diffusion, perfusion, and WB studies [18, 19]. Furthermore, a complete disease assessment in the oncology setting, including detection of metastasis sites, in a single examination helps to reduce the number of patient visits to an imaging service, thereby reducing related costs [20].

**Fig. 1:**
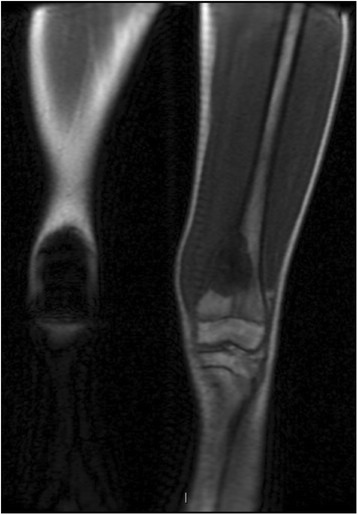
A 15-year-old male patient follow up whole-body MRI examination by bilateral retinoblastoma and osteosarcoma in right femur, in the last exam, the T1-weighted image demonstrated a lesion with low signal intensity in distal left femur. Histological diagnosis of second osteosarcoma

Exposure to ionizing radiation is a major concern in pediatric patients with cancer [7]. However, imaging methods that use ionizing radiation sources, such as X-ray, CT, BS, and PET/CT, are often routinely employed [21, 22]. The risk of tumour development due to ionizing radiation exposure is related to the amount, intensity, and accumulation of the applied radiation over an individual’s life [23]. Newer CT scanners use iterative image reconstruction which reduces radiation exposure, although, exposure still occurs and repeated studies can incur a significant radiation dose for a patient. This risk increases when radiation exposure occurs at younger ages, especially exposure during childhood. In a recent study that examined the risks associated with the use of imaging methods that employ ionizing radiation for diagnostic purposes, an estimated 29,000 new cancer cases were found to be related to the number of CT scans performed in the United States in 2007, with 15% of the estimated cancers associated with scans that were performed on patients younger than 18 years [24, 25]. An advantage of WB MRI is that it can be applied to at-risk populations, including those that may be affected by familial syndromes, to conduct cancer screenings. Advances in genetics have enabled the identification of patients with hereditary syndromes related to the development of neoplasms. The goal of performing WB MRI for cancer screenings is to detect malignancies in their early stages when the effectiveness of treatments and cure rates are optimal [26, 27]. Ideally, screenings should be applied to apparently healthy populations that are at high risk for tumour development. Examples of inherited syndromes that are associated with increased risks of cancer include: multiple endocrine neoplasias I and II (e.g., endocrine tumours), Von Hippel-Lindau syndrome (e.g., renal carcinomas), familial adenomatous polyposis (e.g., colorectal tumours), and Li-Fraumeni syndrome (e.g., various types of tumours including sarcomas) (Fig. 1) [28–31]. Correspondingly, a new screening protocol that involves WB MRI has recently been proposed for patients with Li-Fraumeni syndrome [32].

### Magnetic resonance technique / protocol

To date, there is no overall consensus regarding a WB MRI protocol for children. Typically, T1 and T2 imaging are performed with free-breathing, suspended respiration, or physiological motion control. It is also widely accepted that short tau inversion recovery (STIR) sequences and diffusion add diagnostic value to WB MRI examinations. Depending on a pediatric patient’s age and size, a complete set of images are obtained in a single acquisition (e.g., for infants), or in two or more segmental acquisitions (e.g., for older children and adolescents). The images are subsequently aligned using specific software to enable visualization of the entire body [20, 33]. WB MRI should be performed with high-field (≥1.5 Tesla) equipment with surface and/or body coils. Dedicated pediatric receiver coils are currently being introduced and will progressively have increased availability. Patients should be examined from head to toe in a supine position with their arms parallel to the body and their legs together. Coronal acquisition, which is more rapid than other approaches, is preferred, although at least one sequence (e.g., diffusion) should be acquired in the axial plane to compensate for the limitations associated with coronal acquisition (e.g., for the ribs, sternum, cranium, and spine) [34]. Sagittal acquisition is helpful for evaluating the spine. However, depending on the manufacturer, it is possible to perform axial T1-weighted acquisitions without wasting time. Moreover, synchronization of respiratory and cardiac movements can be achieved with external gates to avoid physiological motion artifacts [5, 33].

Conventional sequences, including T1- and T2-weighted spin-echo sequences, are usually performed without the administration of a paramagnetic contrast medium. Malignant lesions are usually hypointense (low signal intensity) on T1-weighted images (Fig. 1) and have a high signal intensity on T2-weighted images [35].

**Fig. 2:**
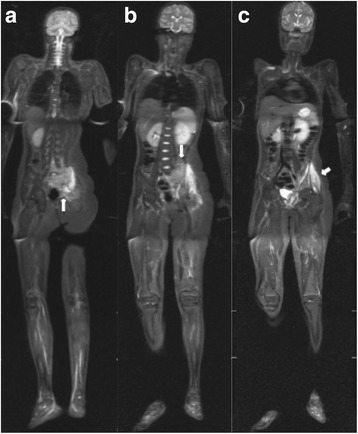
An 18-year-old female patient with a voluminous lesion in the *left* hemi-pelvis (a, *arrow*) and histologically confirmed chondrosarcoma underwent whole-body MRI with a coronal STIR sequence for staging. The examination revealed involvement in the sacrum (**a**, *arrow*) and *left* ilium (**b**, *arrow*) and the presence of soft-tissue components adjacent to marked hyperintensity (**c**, *arrow*). Note the antalgic position, with slight body deviation to the *right*. No other lesion suspicious of malignancy was noted

**Fig. 3:**
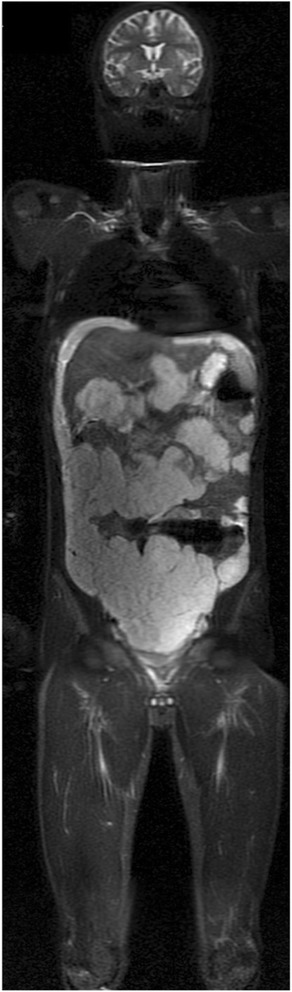
Whole-body MRI with a coronal STIR sequence in a 17-year-old male patient with multiple lesions disseminated in the peritoneal cavity and a histological diagnosis of mucinous adenocarcinoma of high-grade colonic with abdominal implants

The STIR sequence is highly sensitive for the detection of pathologic lesions. Bone marrow lesions, including marrow infiltration from lymphoma, metastases, and tumour-related edema, exhibit high signal intensity on STIR sequences (Figs. 2 and 3). Focal parenchymal lesions can be distinguished by their slightly different signal intensity in STIR sequences, while pathologic lymph nodes cannot be differentiated from normal nodes on the basis of signal intensity. The STIR technique also cannot be used to differentiate benign conditions from malignant neoplastic lesions. The latter limitation restricts the application of STIR in WB MRI in oncologic patients after treatment, since therapy-induced marrow changes, such as edema, necrosis, fibrosis, or red marrow hyperplasia, cannot be differentiated from viable tumours. However, STIR may be very useful in staging pediatric tumours; although, additional clinical experience and data are needed to determine its efficacy [36, 37].

**Fig. 4:**
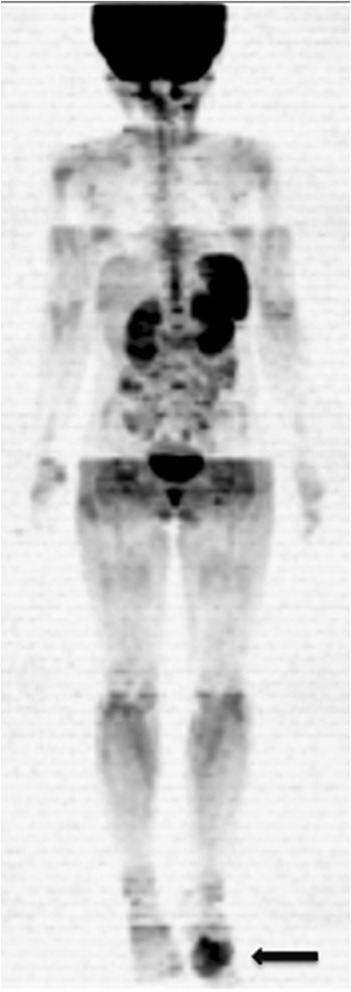
A 9-year-old male patient with a histological diagnosis of osteosarcoma in the left foot (*arrow*) underwent whole-body MRI with diffusion sequences with three-dimensional reconstruction for staging, which showed no other lesion site

Diffusion-weighted MRI sequences are increasingly being employed for WB evaluations of patients with cancer (Fig. 4). These sequences detect the random motion of water molecules, also known as Brownian motion, through biological tissues by detecting the protons in the water molecules. The movement of the water molecules causes a phasic dispersion of proton spin, thereby resulting in signal loss due to diffusion sensitivity. The signal intensity of an object of study is analyzed quantitatively by calculating the absolute apparent diffusion coefficient (ADC, in mm^2^/s) of the object relative to the diffusion of water molecules in the proximal region [16, 38]. This qualitative and quantitative analysis is primarily influenced by the presence of barriers that restrict the diffusion of water molecules in their microenvironment, and this produces imaging contrast between tissues. Thus, the signal intensities and ADCs of different tissues are distinct as a result of their structural characteristics. The restricted diffusion of water molecules that characterises malignant tumours is potentially due to the increased cell density of these tumours, thereby resulting in increased signal intensity in DWI and reduced ADCs, both of which facilitate detection of malignant lesions [16]. However, it should be noted that particularly in children, a high signal in DWI with body background suppression (DWIBS) can be a normal finding in the bony pelvis and lumbar spine. The different types of tissues with high cellularity that are present within these bones contribute to this high signal [39].

Currently, paramagnetic contrast agents can be used in WB MRI examinations, although they are not always indicated. It has been considered that the addition of post-contrast sequences significantly increases examination time and that the behavioral characteristics of lesion enhancement cannot be examined in the arterial, venous, and equilibrium phases simultaneously in WB scans, especially when multiple organs are involved [40]. However, strategies have been developed to address these limitations. For example, the Dixon technique achieves a uniform separation of water and fat that is resistant to large-field inhomogeneities compared to fat suppression by chemical shift selective saturation (CHESS) [41]. Furthermore, when properly implemented, the Dixon technique can be used to acquire either T1-weighted [42] or T2-weighted [43] images within a single breath hold. These rapid Dixon sequences have been successfully incorporated into a WB MRI protocol that is capable of providing multisequence and multiplanar scans, including triphasic (arterial, portal-venous, and equilibrium or delayed) contrast-enhanced imaging of the liver, in approximately 1 h [44]. This Dixon-based WB MRI with multisequence and multiplanar images are also complementary and facilitate high-confidence reading, while multisequence and triphasic contrast-enhanced abdominal imaging is very useful for the detection and characterization of lesions in the liver, an imaging examination that is more commonly performed in adult examinations [44, 45]. On the other hand, however, it is important to consider that administration of paramagnetic contrast agents are associated with some risks, such as those for accidental puncture, allergic reaction, renal failure, and nephrogenic systemic fibrosis [46]. Consequently, the prudent use of gadolinium-based contrast agents to avoid or minimise the risk of nephrogenic systemic fibrosis cannot be overemphasised, as pediatric oncologic patients are more likely to have impaired renal function secondary to anti-cancer therapy [47]. Pediatric patients should also be examined for hepatic lesions following the administration of paramagnetic contrast reagents, despite hepatic lesions being less frequent in children than in adults.

For patients who are unable to receive gadolinium-based contrast agents, ferumoxytol may be a useful MRI contrast agent. Ferumoxytol is an ultrasmall superparamagnetic iron oxide (USPIO) that is comprised of iron oxide particles surrounded by a carbohydrate coat. Initially, this agent was used to treat anemia in patients with chronic renal failure [48]. However, more recently, ferumoxytol has been investigated as an intravenous contrast agent in MRI. The advantages of ferumoxytol include its ability to be administered as a bolus injection, allergic and idiosyncratic reactions with its administration have been limited, and it is not associated with a risk for nephrogenic systemic fibrosis [48, 49]. Femuroxytol also has a long intravascular half-life of 14–15 h [50], and thus, can be used to obtain different types of images. However, there is currently a paucity of data available regarding its use as a paramagnetic contrast agent.

### Clinical indications

WB MRI can be applied for lesion detection/staging, evaluation of treatment response, and follow-up and screening of children with cancer predisposition syndromes.

### Lesion detection / staging

Clinical indications for WB MRI in pediatric patients with cancer depend on the disease type and stage of management. For several types of neoplasms, WB MRI has been shown to be a valid alternative to CT, PET/CT, and scintigraphic studies [20, 35, 47]. Many studies have also shown that WB MRI can be applied at different times during cancer management, including during the screening, staging, response evaluation, and post-therapeutic follow-up stages [5, 20].

The capacity for WB MRI to detect lesions depends on several factors, including the anatomic site, size, histological type, and differentiation grade of the lesions being examined. WB MRI has exhibited good diagnostic accuracy in the staging of a variety of tumours, including both lymphomas and solid tumours [51]. In fact, staging of these neoplasms contributed to the development of this examination technique [5, 51]. Currently, WB MRI can detect lesions present in various anatomical sites, such as the brain, cervical region, thoracic organs, abdomen, bone marrow, and musculoskeletal system. The performance of WB MRI has also been shown to be similar to that of PET/CT in the staging of different cancers, and superior to CT, BS, and scintigraphy with gallium in evaluations of certain osseous and extra-osseous metastases [35, 52, 53].

WB MRI enables a proper assessment of WB bone marrow and the detection of compromised neoplastic sites, including primary tumours and metastases arising from diffusion [37]. However, since normal red marrow impedes diffusion, this may confound disease detection in younger children. Patients with melanoma and Langerhans cell histiocytosis may be evaluated with WB MRI. In fact, the sensitivity, specificity, and accuracy of this method in these cases has been found to similar or superior to those of other methods, including those employing MIBG [20, 54]. WB MRI has also been shown to perform well in the detection of bone metastases, with a higher positive predictive value (94 vs. 76%, respectively) and greater sensitivity (99 vs. 26%, respectively) observed compared with bone scintigraphy [35]. The use of WB MRI is limited in the detection of rib and skull lesions, although the use of respiratory synchronization (triggering) has been shown to reduce the occurrence of motion artifacts, thereby improving the ability of WB MRI to evaluate these anatomic sites [20, 55].

MRI provides different image contrasts that represent specific tissue characteristics. This is important for evaluations of primary tumours and metastases in the brain. Furthermore, if a lesion is detected in the brain or in another part of the body during a WB MRI exam, then a region-specific exam also needs to be conducted. Thus, for patients with Li-Fraumeni syndrome who have an elevated risk of brain tumours, a brain-specific protocol should be added to a WB MRI protocol.

Oncologic patients may undergo multiple MRI, thereby receiving repeated administrations of a gadolinium-based contrast agent. A high signal in the dentate nucleus and globus pallidus on unenhanced T1-weighted images should be cautiously evaluated in these patients, since the signal observed may be a consequence of the number of times that a gadolinium-based contrast material was administered, and not due to the presence of pathologic lesions [56, 57].

Currently, the most important clinical applications of WB MRI in children include the staging of malignant disease and screening for metastatic spread. These applications are particularly relevant in cases involving lymphoma and solid tumours.

#### Lymphoma

**Fig. 5:**
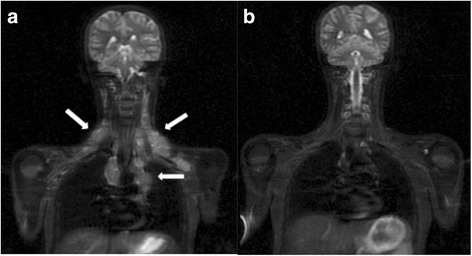
Whole-body MRI with a coronal STIR sequence highlighting the cervical and thoracic regions of a17-year-old female patient with histologically confirmed non-Hodgkin lymphoma. **a** Multiple cervical lymphadenopathies in the supraclavicular and anterior mediastinal regions (*arrows*) were detected at diagnosis. **b** A follow-up examination performed 15 days after chemotherapy showed that the lesions had disappeared

Diagnostic imaging provides important information regarding the staging and response assessment of lymphomas. Recently, a combination of CT and PET was applied to lymphoma staging and evaluations of treatment response [58]. However, both PET and CT involve substantial radiation exposure, and children often undergo several PET/CT examinations during a treatment course. Thus, WB MRI represents a radiation-free alternative for lymphoma staging and follow-up (Fig. 5). Furthermore, when WB MRI and CT were compared in their capacity to provide staging of lymphoma, WB MRI was able to provide disease staging, detect lymph nodes greater than 1.2 cm (with sensitivity and specificity values of 92.0 and 99.9%, respectively), and evaluate the presence or absence of disease spread to bone marrow [58]. In a study of eight children with lymphoma, WB MRI with a coronal STIR sequence was also more sensitive than conventional imaging (e.g., radiography, nuclear medicine studies - bone scintigraphy and gallium scintigraphy - and CT) in detecting bone marrow involvement in the initial stages of disease [15]. Following treatment, however, residual and therapy-induced bone marrow signal abnormalities could not be differentiated from lymphomatous involvement [15].

#### Solid tumours

**Fig. 6:**
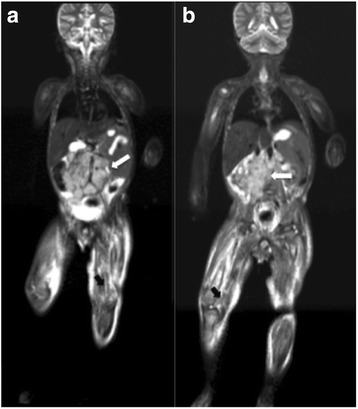
Whole-body MRI with a coronal STIR sequence was performed in a 3-year-old male patient with histologically confirmed neuroblastoma. **a** and **b** The primary lesion appears as an extensive retroperitoneal mass (*white arrows*), and multiple bone metastases (*black arrows*) are present in the femoral

**Fig. 7:**
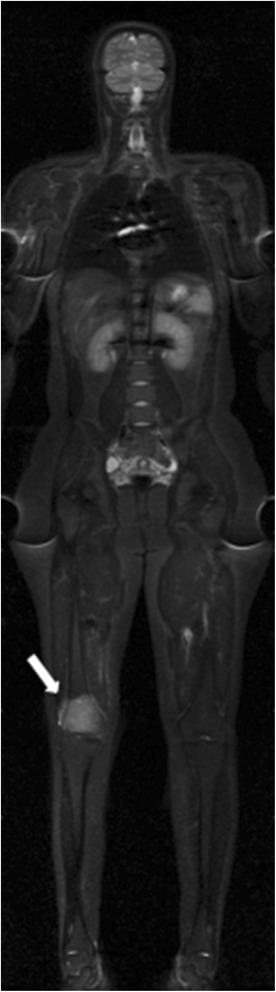
Whole-body MRI with a coronal STIR sequence in a15-year-old male patient with a lesion in the right distal femur and a histological diagnosis of Ewing's sarcoma showed surrounding soft-tissue components (*arrow*), but no other area of abnormal signal intensity suggestive of malignancy

In various pediatric studies, the sensitivity of WB MRI for the detection of distant metastases has been compared with the sensitivities of radiography, CT, conventional MRI, nuclear medicine studies, and PET/CT [53, 54, 59]. Additional studies have suggested that WB MRI is a promising method for the detection of metastases in patients with small cell tumours, and that WB MRI provides at least equivalent information to conventional imaging studies [53, 59]. Neuroblastoma (Fig. 6), primitive neuroectodermal tumour, rhabdomyosarcoma, and Ewing’s sarcoma (Fig. 7) are small round-cell malignancies that have been found to occur in the pediatric population. For the detection of metastases to bone, most investigators have reported a sensitivity of more than 97% for WB MRI, and WB MRI has consistently exhibited a sensitivity comparable to, or greater than, that of skeletal scintigraphy with technetium 99 m (^99m^Tc) medronate disodium [35, 53, 59].

### Evaluation of treatment response and follow-up

**Fig. 8:**
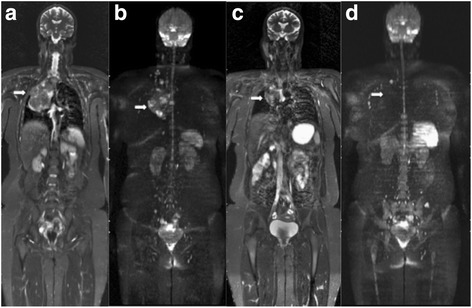
A 16-year-old female patient with primary mediastinal Hodgkin lymphoma (stage III-B) underwent whole-body MRI with STIR (**a**, **c**) and diffusion (**b**, **d**) sequences. **a** and **b** Examinations performed for pre-therapeutic staging showed a voluminous anterior mediastinal lesion (*arrows*). **c** and **d** Post-therapeutic imaging showed the presence of a residual mediastinal lesion with no sign of activity (*arrows*)

WB MRI can be used to evaluate therapeutic response in pediatric oncology patients [51, 60, 61], and the information obtained can be used in combination with Response Evaluation Criteria in Solid Tumors (RECIST) [61]. For example, WB MRI can provide a morphological assessment of target lesions by measuring their major axes according to RECIST, while also providing a functional evaluation of lesions with diffusion sequences. Several studies have described the use of WB MRI to identify partial or complete responses, including increased absolute ADC, after the application of chemo- or radiotherapy to brain tumours, liver tumours, and sarcomas [62, 63]. Similar to CT and PET/CT, WB MRI can also be useful in evaluating significant morphological and functional improvements in lymphomas (Fig. 8), and these are often characterised by an inverse correlation between the tendency toward increased ADC and reduced tumour volume [35, 52]. Furthermore, WB MRI can help distinguish between abnormal scarring and recurrence after therapy [64], thereby enabling the detection of any complications that are related or unrelated to disease or treatment.

### Screening of children with cancer predisposition syndromes

**Fig. 9:**
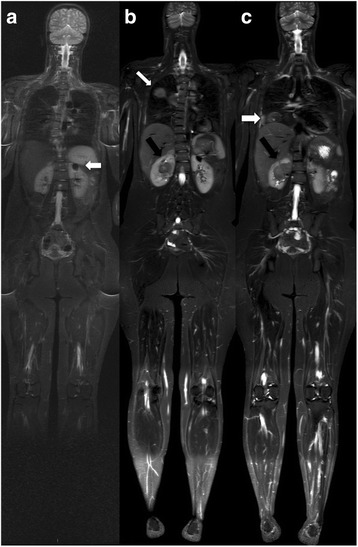
A 20-year-old female patient with Li-Fraumeni syndrome in whom multiple neoplasms had developed since childhood, including lymphoma, soft-tissue sarcomas in the back and thigh, malignant fibrous histiocytoma in the buttock, and adrenal carcinoma. Follow up whole-body MRI examination since 2013, in the last the coronal STIR sequence demonstrated the presence that new lung lesions and kidney nodule. Histological diagnosis suggestive that metastasis of pleomorphic undifferentiated sarcoma in lung and renal cell carcinoma in kidney. Current Whole-body MRI with a coronal STIR sequence demonstrate the presence of multiple lesions in lung (**b**, **c** *white arrows*) and the kidney nodule (**b**, **c** *black arrows*), compared to previous exam which demonstrated only the presence of simple renal cyst (*arrow*), with no other change suggestive of malignancy (**a**)

**Fig. 10:**
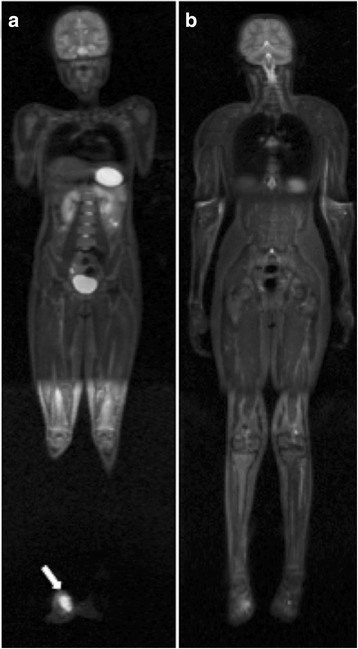
A 16-year-old male patient with histologically confirmed osteosarcoma on the dorsum of the right foot underwent whole-body MRI with a coronal STIR sequence for staging. **a** Note the lesion in the right foot (*arrow*). **b** No skip metastasis or distant lesion was detected

**Fig. 11:**
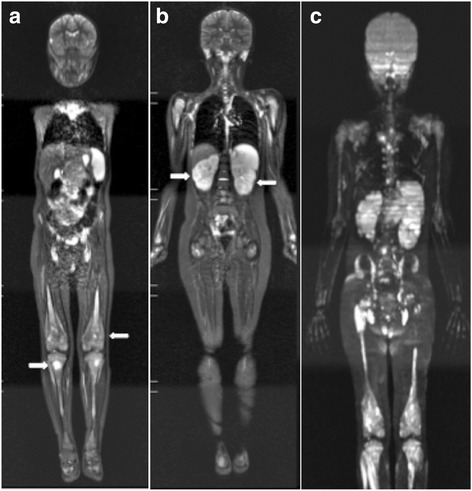
Whole-body MRI was performed in a 6-year-old male patient with acute lymphoblastic leukemia for staging. **a** Diaphyseal and metaphyseal lesions in the distal femoral and proximal tibial regions are represented by hyperintense signals in a coronal STIR sequence (*arrows*). **b** Signs of bilateral renal infiltration by the underlying pathology (*arrows*) were observed. **c** A diffusion sequence demonstrated multiple hyperintense foci, consistent with leukemic infiltration

Cancer predisposition syndromes include a multitude of cancers in which a mode of familial inheritance has been clearly established, although a specific genetic defect may not have been identified [65]. WB MRI has been useful in the screenings of children with cancer predisposition syndromes, and it also has the potential to provide a preclinical diagnosis of any associated tumours. For example, at some institutions, WB MRI is performed annually to screen for tumours in children with Li-Fraumeni syndrome (Fig. 9). Li-Fraumeni syndrome is an autosomal dominant hereditary syndrome that is caused by a loss-of-function mutation in the *TP53* gene and affected individuals have a lifelong increased risk of osteosarcoma (Fig. 10), soft-tissue sarcoma, leukemia (Fig. 11), breast cancer, brain tumour, melanoma, and adrenal cortical tumours [65]. Similarly, individuals with hereditary retinoblastoma (RB) have a very high risk of developing subsequent malignant neoplasms, with osteosarcoma being the most common. When WB MRI screening tests were performed for survivors of hereditary RB, the sensitivity and specificity of detecting subsequent malignant neoplasms was 66.7 and 92.1%, respectively [66].

### Limitations

The use of WB MRI in pediatric oncological clinical practice is limited in some cases. For example, standard contraindications to conventional MRI, such as the presence of metallic body implants or history of claustrophobia, can also preclude the use of WB MRI. Due to the examination time of WB MRI (which can range from 30 min to 1 h), the use of sedation or general anesthesia is typically needed in a significant proportion of pediatric patients, especially those who are young or uncooperative. Thus, the risks associated with these agents must be considered. Immobilization during the examination is also essential for preventing motion artifacts which can impair image acquisition and interpretation of the findings. In addition, physiological artifacts related to respiratory movements, heartbeat, and intestinal peristalsis can impair image acquisition [67]. To minimise the occurrence of these artifacts, multi-channel equipment, body coils, and parallel imaging can be employed. Furthermore, single-shot acquisition, the use of presaturation bands in the anterior body, and mechanisms of synchronization with respiratory and cardiac movements can reduce the time needed for an examination and facilitate the acquisition of highquality images [67, 68]. Finally, the occurrence of false-positive results is another limiting factor in the use of WB MRI. For example, inflammatory abnormalities, infections, and even benign lesions such as simple cysts or vascular lesions have been found to simulate malignant lesions [67].

## Conclusion

Currently, WB MRI is able to provide total body coverage, high tissue contrast, and good spatial resolution without the use of radiation. Moreover, the ability to obtain relevant morphological and functional information in a single examination represents a key advantage of this method in the management of pediatric oncology patients.

## Abbreviation

^18^F-FDG: ^18^F-fluorodeoxyglucose
^99m^Tc: Technetium 99m
ADC: Absolute apparent diffusion coefficient
ADC: Absolute apparent diffusion coefficient
BS: Bone scintigraphy
CHESS: Chemical shift selective saturation
CT: Computed tomography
DWI: Diffusion- weighted imaging
DWIBS: Diffusion-weighted imaging with body background suppression
MIBG: Metaiodobenzylguanidine
MRI: Magnetic resonance imaging
PET/CT: Positron emission tomography/computed tomography
RB: Retinoblastoma
RECIST: Response Evaluation Criteria in Solid Tumors
STIR: Short tau inversion recovery
USPIO: Ultrasmall superparamagnetic iron oxide
WB: Whole body
